# Data-Driven Design and Optimisation of Eutectic-Assisted Alumina Ceramics Using RSM and Symbolic Regression

**DOI:** 10.3390/ma19214561

**Published:** 2026-10-29

**Authors:** Salman Saeidlou

**Affiliations:** Sustainable Engineering and the Built Environment, Canterbury Christ Church University, Canterbury CT11QU, UK; salman.saeidlou@canterbury.ac.uk

**Keywords:** alumina ceramics, eutectic additives, liquid-phase sintering, response surface methodology, symbolic regression, genetic programming, process optimisation, mechanical properties

## Abstract

Lowering the processing temperature of alumina while retaining mechanical performance requires control of additive chemistry and powder processing. This study examines CaO–SiO_2_–Al_2_O_3_-assisted alumina through a 27-condition dataset, response surface methodology (RSM) and symbolic regression using GPTIPS 2.0. Sintering temperature, coarse-alumina mass fraction, lubricant content and additive content are the input variables; flexural strength and density are performance responses, while shrinkage is treated as a dimensional response. The highest tabulated strength is 173.52 MPa at 1550 °C, with a density of 3.610 g/cm^3^. GPTIPS evaluation-subset variation explained is 89.55%, 87.39% and 81.24% for strength, density and shrinkage, respectively. These statistics describe model selection rather than independent predictive validation. A comparison with published reduced-cubic RSM models gives strength and density R^2^ values of 0.9825 and 0.9934, whereas full-quadratic models yield lower adjusted R^2^ values. The results demonstrate the influence of model specification on fitted agreement and distinguish the maximum tabulated response from a constrained literature optimum. The combined analysis provides a basis for assessing processing–property relationships while recognising the dimensional and service-performance trade-offs associated with liquid-phase-assisted sintering.

## 1. Introduction

Alumina is widely used in refractory, abrasive, electronic and biomedical applications because of its hardness, chemical stability and electrical insulation [1]. Optical applications also include carbon-doped alumina dosimeters [2]. Its thermal and mechanical performance depends on purity, porosity, grain structure and service conditions; thermal-shock resistance should therefore not be assumed to be uniformly high.

Alumina components are typically fabricated through the compaction or injection of alumina powders into moulds with the desired geometry, followed by sintering of the resulting green bodies at elevated temperatures. Consequently, significant attention has been directed in recent research towards reducing the sintering temperature without causing substantial degradation in the resulting ceramic properties [3,4,5]. The addition of low-melting-point additives, such as eutectic phases capable of forming intergranular liquid layers, has been shown to facilitate the sintering and bonding of ceramic particles at reduced temperatures [3]. These liquid-phase layers are able to wet the ceramic surfaces and infiltrate the interparticle spaces, leading to particle rearrangement that promotes ceramic densification. Furthermore, the surface tension associated with the liquid phase contributes to enhanced densification and assists in the elimination of pores within the ceramic matrix [6].

Liquid-phase-forming additions assist alumina densification, with the outcome depending on additive chemistry, quantity and processing conditions [3,4,5]. The CaO–Al_2_O_3_–SiO_2_ phase equilibria provide a basis for selecting compositions with low liquid-formation temperatures [7].

In addition to the type and amount of the liquid phase, several other parameters—such as the particle size and size distribution of alumina, the type and concentration of the lubricant agent, the sintering temperature, and the soaking time—have also been shown to influence the final properties of sintered components. The conventional “one factor at a time” approach, which is often employed for optimising these variables, has been found to be both time-consuming and labour-intensive, as it requires a large number of experimental trials. Moreover, this method fails to account for the potential interactions among the influencing factors, rendering it inadequate for effective optimisation [8,9]. Therefore, the application of statistical methods for experimental design has been deemed necessary to reduce the number of required trials and, consequently, to minimise both time and cost. Among these methods, RSM has been widely employed. RSM is a statistical technique capable of modelling the relationship between multiple factors at varying levels and the corresponding responses, typically by fitting a polynomial equation that characterises these interactions [8].

Assuming that all factors are measurable, the response surface may be represented by the following general equation:

*y*
= *f*(*x*_1_*͵x*_2_*͵x*_3_*͵*…) (1)

In this expression, *y* denotes the response, while *x*_1_ to *x_n_* represent the process variables or input factors influencing the response [10]. In essence, RSM comprises three main stages: (1) the design and execution of experiments, (2) the development of the RSM model using regression analysis, and (3) the optimisation of the response [11].

In recent years, considerable attention has been directed towards the application of RSM by researchers. For instance, RSM was employed by Li et al. [8] to optimise the relative density and bending strength of sintered CaO-partially stabilised zirconia (CaO-PSZ), using soaking time, sintering temperature, and heating rate as process variables. Similarly, Sktani et al. [9] applied this method to model and optimise the toughness and hardness of zirconia-toughened alumina (ZTA) ceramics, considering chemical composition, sintering time, and temperature as input factors. In addition, numerous studies have been conducted on the modelling and optimisation of sintering processes for materials such as Si_3_N_4_–SiC composites [12], titanium powder [10], high-speed steels (HSS) [13], and zirconia-toughened alumina (ZTA) composites [14].

Keramat and Hashemi [15] previously investigated the optimisation of liquid-phase sintering of alumina ceramics using central composite design (CCD) and response surface methodology (RSM). Building upon their published experimental dataset, the present study extends the analytical framework through genetic programming-based symbolic regression (GPTIPS 2.0) [16], incorporating sintering shrinkage as an additional response variable and comparing alternative empirical modelling approaches. The objective is to further investigate nonlinear relationships between processing parameters and ceramic properties, providing complementary insights beyond the previously reported RSM-based optimisation.

Recent work illustrates the range of available processing routes. Garanin et al. [17] combined intensive milling and a CuO–TiO_2_–Nb_2_O_5_ additive to obtain ZTA at 1050 °C. Tang et al. [18] investigated Dy_2_O_3_–Al_2_O_3_–SiO_2_ glass infiltration into dense alumina, while Stosz et al. [19] combined ternary additives with spark plasma sintering. These studies show why additive chemistry must be considered together with particle size, pressure and thermal history when comparing performance.

## 2. Materials and Methods

### 2.1. Synthesis of the CaO–SiO_2_–Al_2_O_3_ Eutectic Phase

The synthesis was carried out through precise stoichiometric mixing and thermal processing. In this manner, to obtain the eutectic composition within the CaO–SiO_2_–Al_2_O_3_ system, high-purity starting materials were employed, including calcium carbonate (CaCO_3_, ~99% purity, ~600 nm), silicon dioxide (SiO_2_, ~99% purity, ~7.8 μm), and kaolin (Al_2_Si_2_O_5_(OH)_4_, ~99% purity, ~1.8 μm). The powders were accurately weighed in stoichiometric proportions corresponding to a eutectic point with a melting temperature of approximately 1270 °C, as shown in Table 1. Initial mixing was performed via planetary ball milling in distilled water for 6 h using zirconia media to avoid contamination. The resulting slurry was dried at 110 °C for 12 h, ground to a fine powder, and calcined at 950 °C for 4 h in air to ensure complete decomposition of carbonate components and to initiate solid-state reactions. A subsequent high-temperature treatment at 1350 °C for 2 h was conducted to promote the formation of the eutectic phases anorthite, gehlenite, and pseudo-wollastonite [7].

Table 2 describes four additive heat treatments: 1200 and 1250 °C, each held for 4 or 8 h. The heat treatments at 1200 and 1250 °C were conducted below the nominal eutectic melting temperature of approximately 1270 °C, promoting solid-state reactions and the formation of the desired crystalline phases. Subsequent sintering of the alumina composites at higher temperatures facilitated liquid-phase formation, contributing to particle rearrangement and densification. The reported processing also includes attrition milling with zirconia media (ball-to-material mass ratio approximately 15:1, 800 rpm, 1 h), compaction at 10 MPa into 18 mm × 3 mm bodies, and pulverisation through a 120-mesh screen before XRD. XRD was conducted using a Bruker D8-ADVANCE with Cu Kα radiation at 40 kV and 40 mA.

Differential scanning calorimetry (DSC) was used to characterise the thermal behaviour of the G1–G4 additive preparations. The resulting thermograms were examined to assess differences in thermal response associated with the additive heat-treatment conditions. The DSC results are presented and discussed in Section 3.2.

### 2.2. Composite Preparation and Mechanical Testing

The fixed-composition additive comparison and the variable-factor RSM matrix are described separately. In the reported preliminary comparison, alumina powder (~99.5% purity; average particle size ~11.1 μm) was mixed with each G1–G4 additive at 93.5:6.5 by mass, followed by calcium stearate addition (reported as 1 wt%). The mixtures were ball milled before compaction. G0 denotes the comparison mixture prepared directly from CaCO_3_, SiO_2_ and kaolin without presynthesis of the additive; it is not an additive-free alumina control.

**Figure 1:**
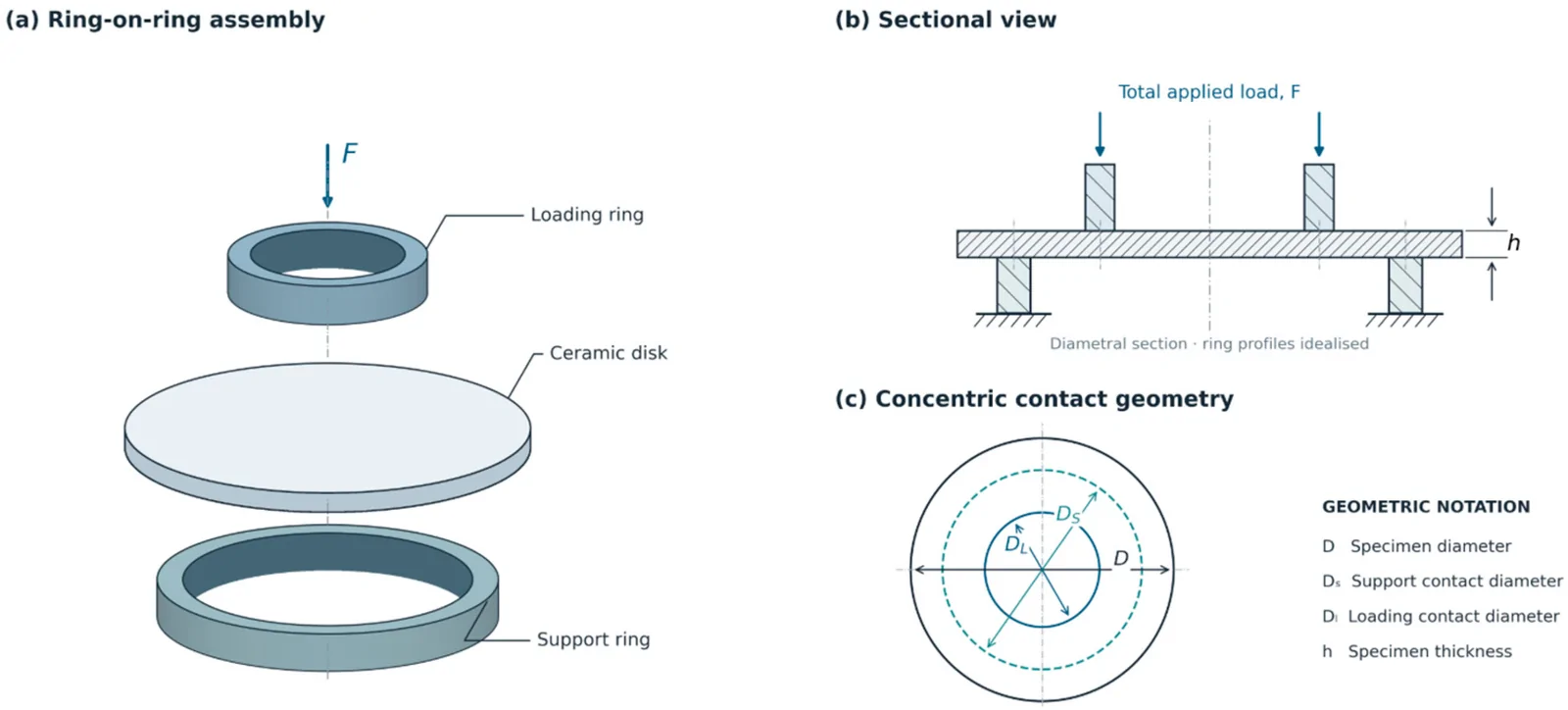
Ring-on-ring equibiaxial flexural testing: (**a**) exploded assembly; (**b**) diametral loading section; and (**c**) concentric contact geometry.

For the preliminary comparison, the reported green specimens were disks of 18 mm diameter and 3 mm thickness, pressed at 40 MPa and sintered at 1400 °C for 4 h in air. The RSM experimental design, presented in Table 3, investigated a wider sintering temperature range of 1400–1600 °C, together with variations in coarse-alumina fraction, lubricant content and eutectic additive content, to evaluate their effects on the mechanical and physical properties of the sintered ceramics. Ring-on-ring testing used a Santam universal testing machine at a crosshead speed of 1 mm/min [20]. The test configuration and concentric loading geometry are illustrated in Figure 1. For the preliminary comparison, at least ten specimens were tested for each composition to assess the mechanical performance of the sintered ceramics. The RSM analysis was conducted using the experimental response values presented in Table 3. Ring-on-ring loading measures specimen equibiaxial flexural strength, not an isolated interfacial bond strength. ASTM C1499-19 (2024) [21] provides the relevant test framework. The circular-disk expression below is also given by Keramat and Hashemi [15] and is included to make the load-to-stress conversion explicit:

(2) $$\sigma_{f}=\frac{3F}{2\pi h^{2}}\left[\left(1-\nu \right)\frac{D_{S}^{2}\;-\;D_{L}^{2}}{2D^{2}}+\left(1+\nu \right)\ln\left(\frac{D_{S}}{D_{L}}\right)\right]$$

Here *F* is the fracture load, *h* and *D* are the measured fired-specimen thickness and diameter, *DS* is the support-ring diameter, *DL* is the loading-ring diameter, and ν is Poisson’s ratio. Loads in N and dimensions in mm give stress in MPa. The expression assumes an appropriate concentric-ring geometry and plate response; contact friction, alignment, warpage and large deflection require assessment.

**Table 3:** Dataset organised by central composite design point type for the present modelling analysis. Coded inputs z_1_–z_4_ represent sintering temperature, coarse-alumina mass fraction, lubricant content and additive content, respectively; conversions to physical units are defined in Section 2.3. Processing inputs, flexural strength and density data are taken from Keramat and Hashemi [15].

Design Group	Source Run	z_1_	z_2_	z_3_	z_4_	Flexural Strength (MPa)	Density (g/cm^3^)	Shrinkage (%)
Factorial	1	+1	+1	−1	−1	113.63	3.319	28.34
Factorial	4	+1	+1	+1	−1	138.04	3.471	29.61
Factorial	7	+1	−1	+1	+1	173.52	3.610	29.04
Factorial	8	−1	−1	+1	+1	114.55	3.412	28.66
Factorial	14	+1	+1	+1	+1	132.11	3.459	27.48
Factorial	15	+1	+1	−1	+1	148.07	3.522	32.37
Factorial	16	−1	+1	+1	−1	130.54	3.346	29.68
Factorial	17	−1	−1	−1	−1	162.00	3.528	29.12
Factorial	18	−1	+1	−1	−1	141.75	3.476	36.41
Factorial	19	−1	+1	−1	+1	146.61	3.504	29.90
Factorial	20	+1	−1	−1	+1	113.33	3.289	23.43
Factorial	22	+1	−1	+1	−1	171.03	3.571	30.95
Factorial	23	+1	−1	−1	−1	113.87	3.346	30.16
Factorial	24	−1	−1	−1	+1	123.03	3.378	34.89
Factorial	25	−1	−1	+1	−1	105.00	3.225	29.08
Factorial	26	−1	+1	+1	+1	125.17	3.411	29.28
Axial	2	0	+2	0	0	98.74	3.197	20.91
Axial	3	0	0	0	−2	143.38	3.494	28.92
Axial	5	0	−2	0	0	132.13	3.448	28.45
Axial	9	0	0	+2	0	142.78	3.471	29.24
Axial	12	0	0	0	+2	150.60	3.521	30.13
Axial	13	−2	0	0	0	159.05	3.512	30.17
Axial	21	+2	0	0	0	111.32	3.307	23.18
Axial	27	0	0	−2	0	131.03	3.417	28.77
Centre	6	0	0	0	0	145.53	3.471	27.70
Centre	10	0	0	0	0	161.56	3.525	30.27
Centre	11	0	0	0	0	146.03	3.499	28.67

### 2.3. Method

The present study employs a four-factor central composite design (CCD) dataset comprising 27 experimental conditions to investigate processing–property relationships in eutectic-assisted alumina ceramics. The processing parameters, flexural strength and density values are derived from the experimental dataset published by Keramat and Hashemi [15]. These data are reanalysed using full-quadratic least-squares models and genetic programming-based symbolic regression (GPTIPS 2.0), while the previously published reduced-cubic RSM models provide a comparative benchmark. Sintering shrinkage is incorporated as an additional response to extend the evaluation of ceramic processing behaviour.

Symbolic regression using GPTIPS 2.0 [16] was used as a complementary empirical modelling approach. Agreement between RSM and GPTIPS fitted to the same measurements does not independently validate either the measurements or the proposed sintering mechanisms.

The physical input variables were:

- x1: Sintering temperature (°C).
- x2: Coarse-alumina mass fraction in the alumina powder blend (%).
- x3: Lubricant content (wt%).
- x4: Eutectic phase content (wt%).

Table 3 presents the processing inputs as dimensionless coded variables: z_1_ = (T − 1500)/50, z_2_ = (C − 50)/25, z_3_ = (L − 1)/0.5 and z_4_ = (E − 6.5)/1, where T is sintering temperature in °C, C is coarse-alumina mass fraction in mass%, and L and E are lubricant and additive contents in wt%, respectively. The same coding is used for the quadratic analysis in Appendix A and the literature-model reconstruction in Appendix B. Responses are retained in physical units. Rows are grouped as factorial, axial and centre points; this presentation order does not alter the source run identifiers or the input–response pairings used in the analyses.

The dataset comprising 27 samples was randomly divided into a training set (70%) and a test set (30%). Each response of fracture strength, density, and shrinkage was modelled separately. The GPTIPS configuration included the following:

- Function set: {+, −, ×, square, sqrt, exp}.
- Population size: 220 individuals.
- Number of generations: 120.
- Maximum tree depth: 4.
- Maximum number of genes: 5.
- Selection method: Tournament (size = 4).
- Fitness function: Root Mean Squared Error (RMSE).

The best evolved models were selected based on their predictive performance on the test set and their interpretability. Parity plots were generated to compare the predicted and actual values. The relative importance of genes and the structure of symbolic expressions were analysed to gain physical insights into the governing mechanisms. In this way, the Pseudo code shown in Algorithm 1 explains the procedure.
**Algorithm 1.** Explanation of the procedure employed to symbolically model the variation in fracture strength, density, and sintering shrinkage with Sintering_Temperature, Alumina_Particle_Size, Lubricant_Content, Glass_Phase_Content.**BEGIN****1. LOAD dataset**   INPUT variables:      X = [Sintering_Temperature, Alumina_Particle_Size, Lubricant_Content, Glass_Phase_Content]   OUTPUT variables:     Y1 = Fracture_Strength     Y2 = Density     Y3 = Sintering_Shrinkage**2. INITIALIZE GPTIPS configuration**   SET function_set = {+, −, ×, square, sqrt, exp}   SET population_size = 220   SET generations = 120   SET max_tree_depth = 4   SET max_genes = 5   SET selection_method = Tournament   SET tournament_size = 4   SET fitness_function = RMSE**3. FOR** each target_variable in {Y1, Y2, Y3} DO**a. CREATE** a GPTIPS model     SET gmodel.input = X    SET gmodel.target = target_variable**b. CONFIGURE** gmodel parameters:    gmodel.funcSet = function_set    gmodel.popSize = population_size    gmodel.genCount = generations    gmodel.maxDepth = max_tree_depth    gmodel.maxGenes = max_genes    gmodel.selection = ‘tournament’    gmodel.tournamentSize = tournament_size    gmodel.fitnessFunction = ‘RMSE’**c. RUN** symbolic regression using GPTIPS    gmodel = GPTIPS_RUN(gmodel)
**d. EXTRACT** best evolved model:    best_model = gmodel.bestIndividual**e. OUTPUT** best_model expression and performance metrics**   END FOR****4. DISPLAY** symbolic models for all three outputs:   - Fracture Strength model   - Density model   - Sintering Shrinkage model**END**

## 3. Results and Discussion

### 3.1. XRD

**Figure 2:**
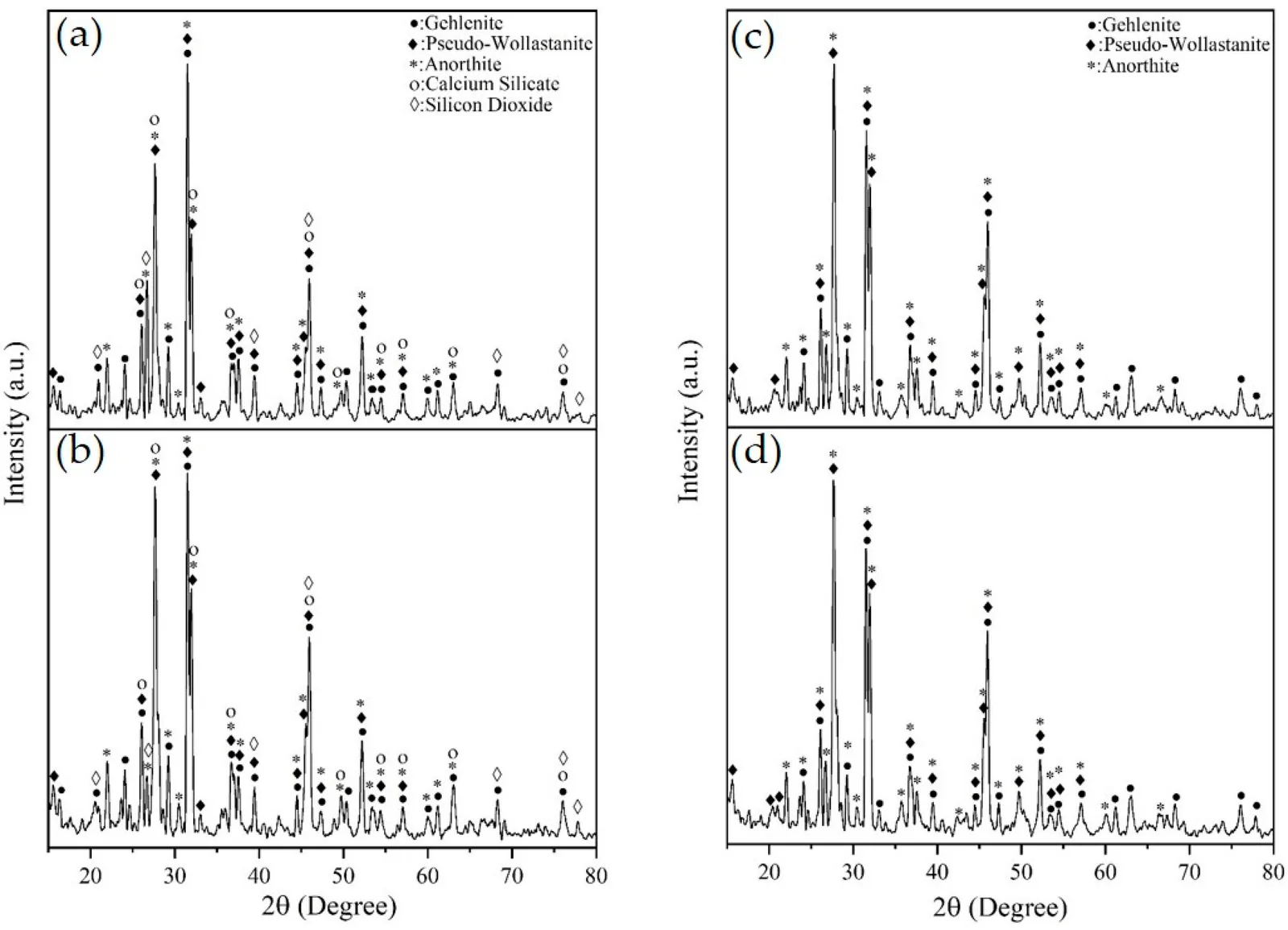
XRD patterns of the CaO–SiO_2_–Al_2_O_3_ additive after heat treatment: (**a**) G1, 1200 °C for 4 h; (**b**) G2, 1200 °C for 8 h; (**c**) G3, 1250 °C for 4 h; and (**d**) G4, 1250 °C for 8 h. The open-diamond symbol denotes α-quartz (SiO_2_).

Figure 2 shows the XRD patterns associated with the G1–G4 heat treatments. The principal phase assignments are gehlenite, anorthite and pseudowollastonite. The residual silica assignment is expressed as α-quartz (SiO_2_), distinguishing the crystalline polymorph from the generic oxide composition. The patterns provide qualitative phase information; phase fractions and glass content are not quantified by this comparison.

### 3.2. Thermal Behaviour and Liquid-Phase Formation

**Figure 3:**
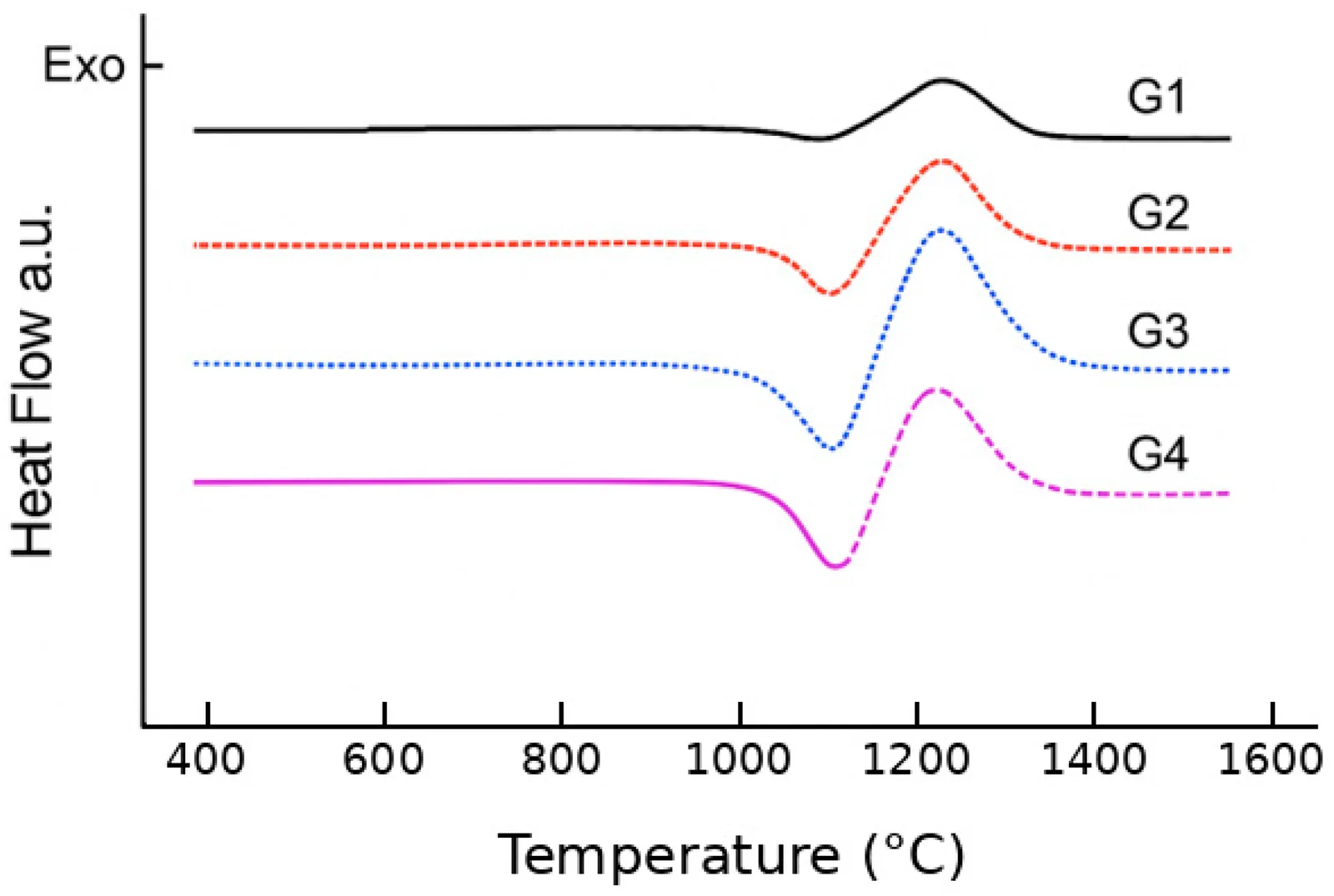
DSC thermograms of the G1–G4 additive preparations defined in Table 2. Heat flow is expressed in arbitrary units; exothermic heat flow is directed upwards.

Figure 3 compares the DSC thermograms of G1–G4. The traces exhibit a downward excursion followed by an upward peak, with exothermic heat flow directed upwards. The downward feature is less pronounced for G1 than for G2–G4. These differences indicate that the additive preparation conditions affect the subsequent thermal response. Peak shape alone does not establish phase identity, reaction completion or a quantitative ranking of additive homogeneity.

Solid-state formation of the additive and liquid formation during composite sintering are separate stages. The 1200 and 1250 °C treatments lie below the nominal eutectic temperature of approximately 1270 °C [15], whereas the 1400–1600 °C composite-sintering interval lies above it. Thus, the lower-temperature preparation treatments are consistent with solid-state reaction, followed by liquid-phase-assisted densification during composite sintering.

The low-temperature liquid can promote wetting, particle rearrangement and pore elimination, while the final microstructure depends on composition, particle size and thermal history [3,4,5,6,7]. The nominal eutectic temperature is a literature-based composition benchmark and is not presented as a DSC onset or peak measured in this study.

### 3.3. Response Surface Analysis and Processing Benchmarks

The CCD comprises four processing factors at five coded levels (−2, −1, 0, +1 and +2). The analysis extends the previously published dataset [15] by examining the response trends using full-quadratic RSM and symbolic regression, with the published reduced-cubic models serving as a benchmark. The original strength and density measurements are retained to enable direct comparison between modelling approaches, while sintering shrinkage is considered as an additional response for evaluating dimensional changes during ceramic processing.

The powder-blend variable is C = 100 mc/(mc + mf), where mc and mf are the masses of coarse (mean size 11.1 μm) and fine (mean size 4.2 μm) alumina, respectively. Thus C = 25% means 25 mass% coarse and 75 mass% fine alumina within the alumina fraction, excluding lubricant and additive. C = 0% and 100% denote fully fine and fully coarse alumina. This variable is a blend mass fraction, not a particle diameter or a percentile of a particle-size distribution.

The design comprises 27 design points, consistent with the CCD count in Equation (3):

*n_e_* = 2*^k^* + 2*k* + *l*, (3)

Here *k* = 4 is the number of factors and *l* = 3 is the number of centre-point runs. Table 3 groups the 16 factorial, eight axial and three centre-point runs separately. The centre points retain source run identifiers 6, 10 and 11.

Ordinary least squares were used to fit a full-quadratic model containing four linear terms, four squared terms and six two-factor interactions. Appendix A gives the coefficients and conditional ANOVA for all three responses. The R^2^ values are 0.612, 0.594 and 0.408 for strength, density and shrinkage, respectively; adjusted R^2^ values are 0.160, 0.121 and −0.283, with overall model *p*-values of 0.302, 0.350 and 0.827. The negative adjusted R^2^ for shrinkage indicates that the quadratic basis offers little explanatory benefit after accounting for its parameter count. These statistics apply to the specified quadratic models.

**Figure 4:**
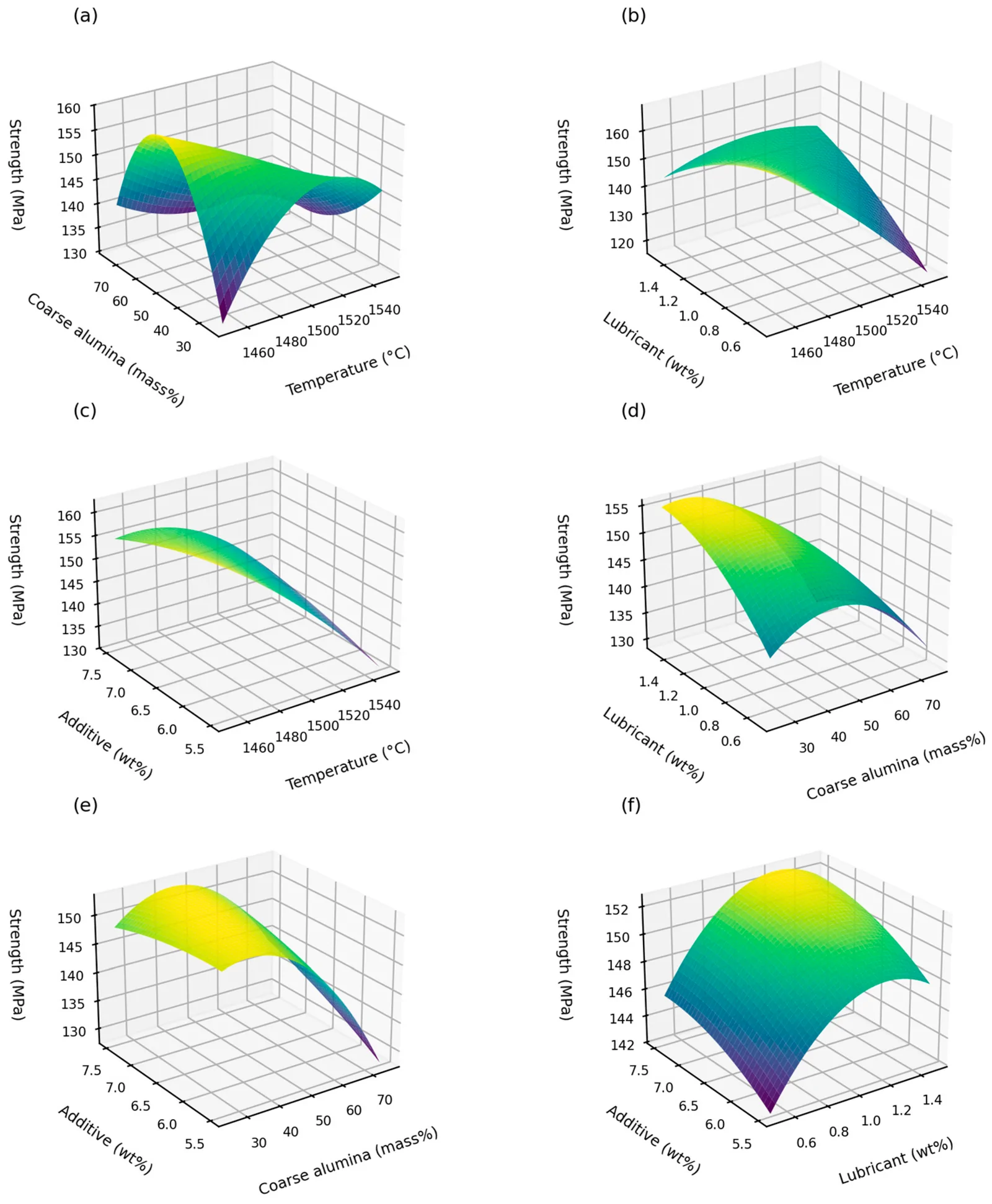
Calculated strength surfaces from the reconstructed literature reduced-cubic model, using the coefficients in Appendix B. Panels (**a**–**f**) show factor pairs (T,C), (T,L), (T,E), (C,L), (C,E) and (L,E), respectively. The remaining factors are held at their centre values.

**Figure 5:**
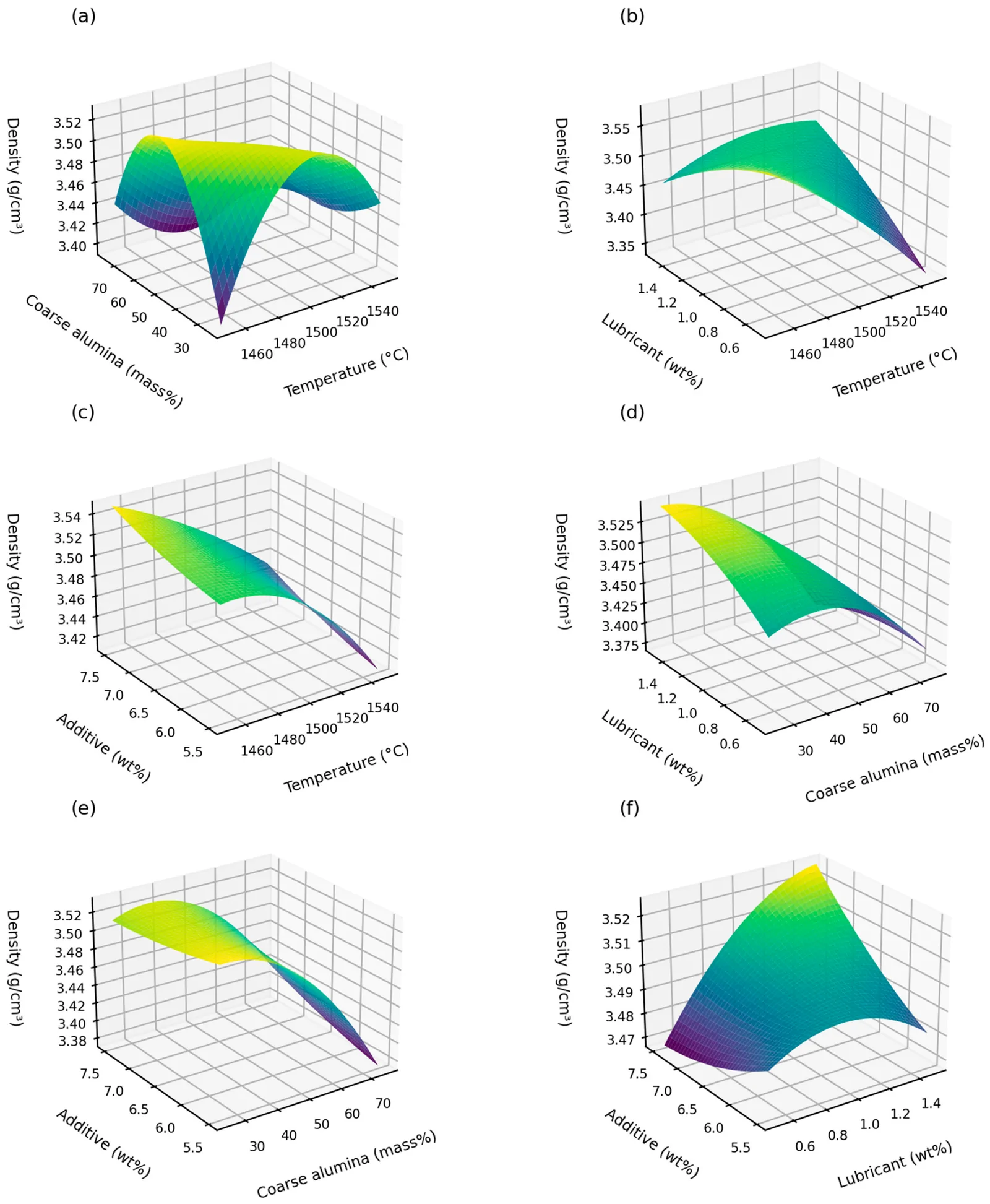

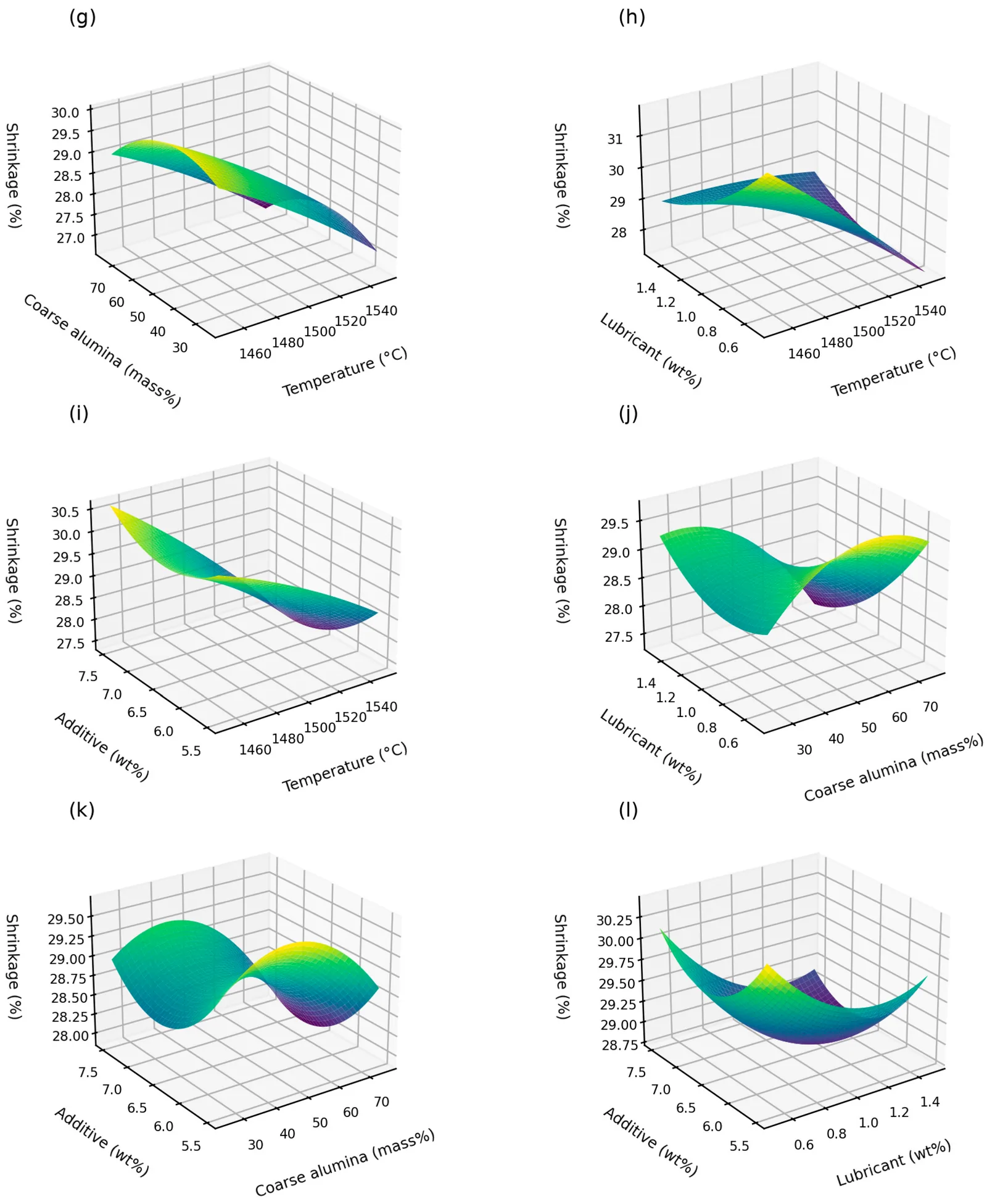
Calculated response surfaces for density (**a**–**f**), obtained from the reconstructed literature reduced-cubic model using Appendix B coefficients, and sintering shrinkage (**g**–**l**), obtained from the quadratic model using Appendix A coefficients: (**a**,**g**) sintering temperature and coarse-alumina mass fraction; (**b**,**h**) sintering temperature and lubricant content; (**c**,**i**) sintering temperature and additive content; (**d**,**j**) coarse-alumina mass fraction and lubricant content; (**e**,**k**) coarse-alumina mass fraction and additive content; and (**f**,**l**) lubricant content and additive content. In each panel, the remaining two factors are held at their centre values.

For comparison, the 23-coefficient reduced-cubic basis specified in [15] was reconstructed from the published rounded values. Appendix B reports its retained terms, coefficients and model-level ANOVA. Strength and density R^2^ values are 0.9825 and 0.9934, with adjusted R^2^ values of 0.8864 and 0.9571. Figure 4 and Figure 5 illustrate the reconstructed literature strength and density surfaces and the quadratic shrinkage surface. Each panel varies two coded inputs between −1 and +1 while holding the other two at their centre values. The reduced-cubic fits have four residual degrees of freedom; their high fitted agreement is not an independent validation result.

The maximum tabulated strength is 173.52 MPa at 1550 °C, 25% coarse alumina, 1.5 wt% lubricant and 7.5 wt% additive (source run 7 in Table 3); density and shrinkage are 3.610 g/cm^3^ and 29.04%. This tabulated maximum differs from the constrained optimum reported in [15], which is 1533 °C at the same remaining factor settings. The source predicts 172.49 MPa and 3.566 g/cm^3^ and reports confirmation measurements of 163.15 MPa and 3.767 g/cm^3^. These extracted literature values provide a processing benchmark. Appendix B summarises the source objective settings and distinguishes prediction from confirmation.

Comparisons with other processing routes are given in Table 4. Lower furnace temperature alone is not a complete measure of energy efficiency; milling, dwell time, pressure, additive preparation and final properties also matter. Different matrices, flaw populations and test geometries preclude a direct ranking of strength values across studies.

Residual grain-boundary glass can promote densification but may also permit viscous deformation and grain-boundary sliding at elevated temperature, alter dielectric loss, and change thermal stresses. These are composition-dependent risks, not measured outcomes of the present dataset. Glass-containing alumina can also show improved damage recovery under particular conditions [18]. No service-temperature limit can be assigned here without glass-transition/softening data, creep tests, dielectric measurements over frequency and temperature, and thermal-shock testing. Post-sintering crystallisation annealing is a possible future treatment, but must be evaluated for porosity, residual stress and mechanical degradation rather than presumed beneficial.

### 3.4. Symbolic Regression-Based Modelling of Ceramic Properties

GPTIPS 2.0 was used to explore nonlinear relationships between the four processing factors and the three responses. The resulting training and evaluation plots characterise the empirical model fit and the variation explained during model selection.

The 70:30 partition separates fitting and evaluation observations within the 27-row dataset. Because the evaluation subset also contributes to model selection, its reported performance is interpreted as an internal evaluation measure. The small dataset and repeated centre conditions limit the strength of conclusions about generalisation.

**Figure 6:**
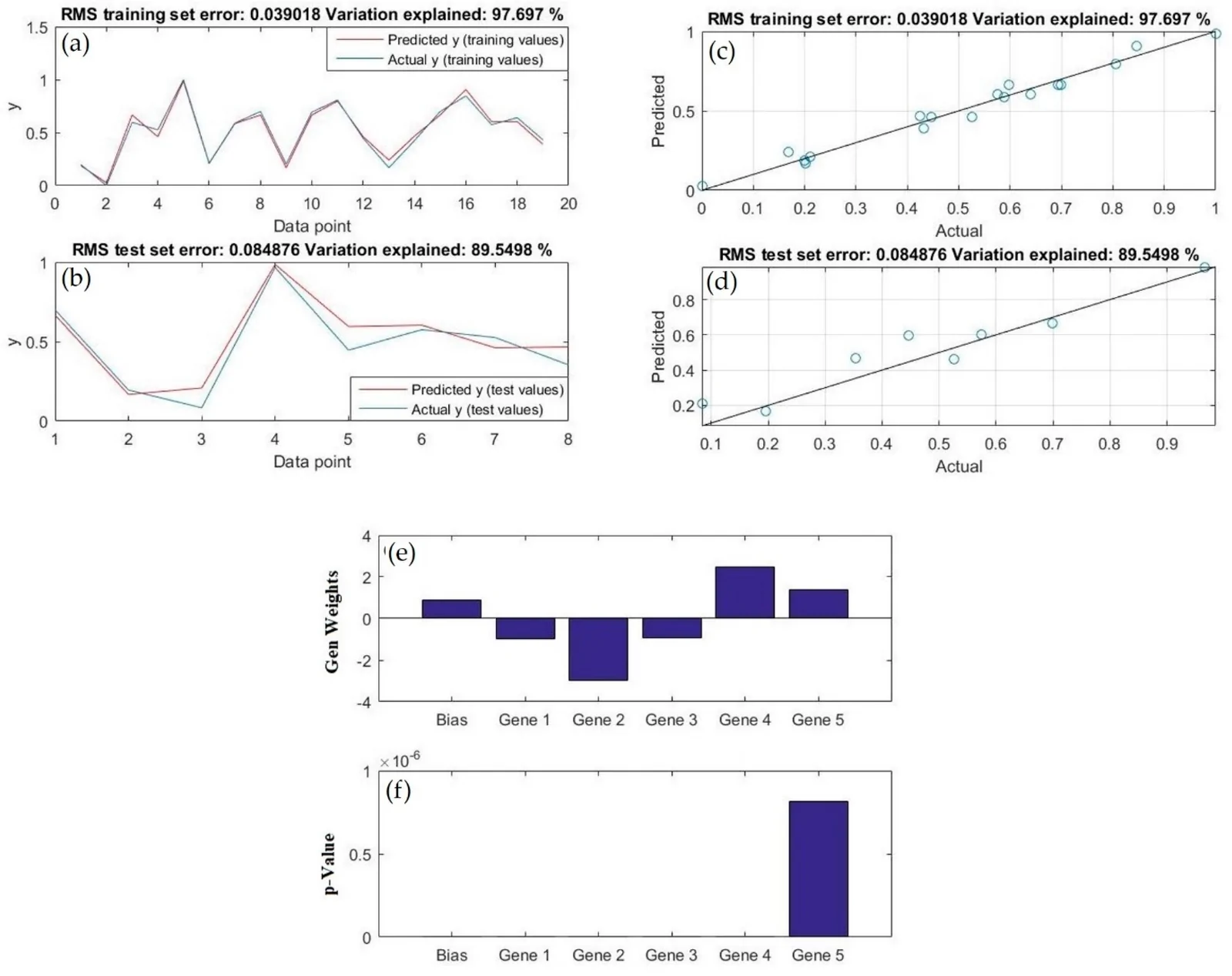
GPTIPS strength outputs: response traces for (**a**) training and (**b**) evaluation observations; predicted-versus-observed values for (**c**) training and (**d**) evaluation observations; and (**e**) weighted gene contributions and (**f**) reported gene *p*-values. The plot labels “test” refer to the model-selection evaluation subset. Response coordinates are on the plotted transformed scale.

**Figure 7:**
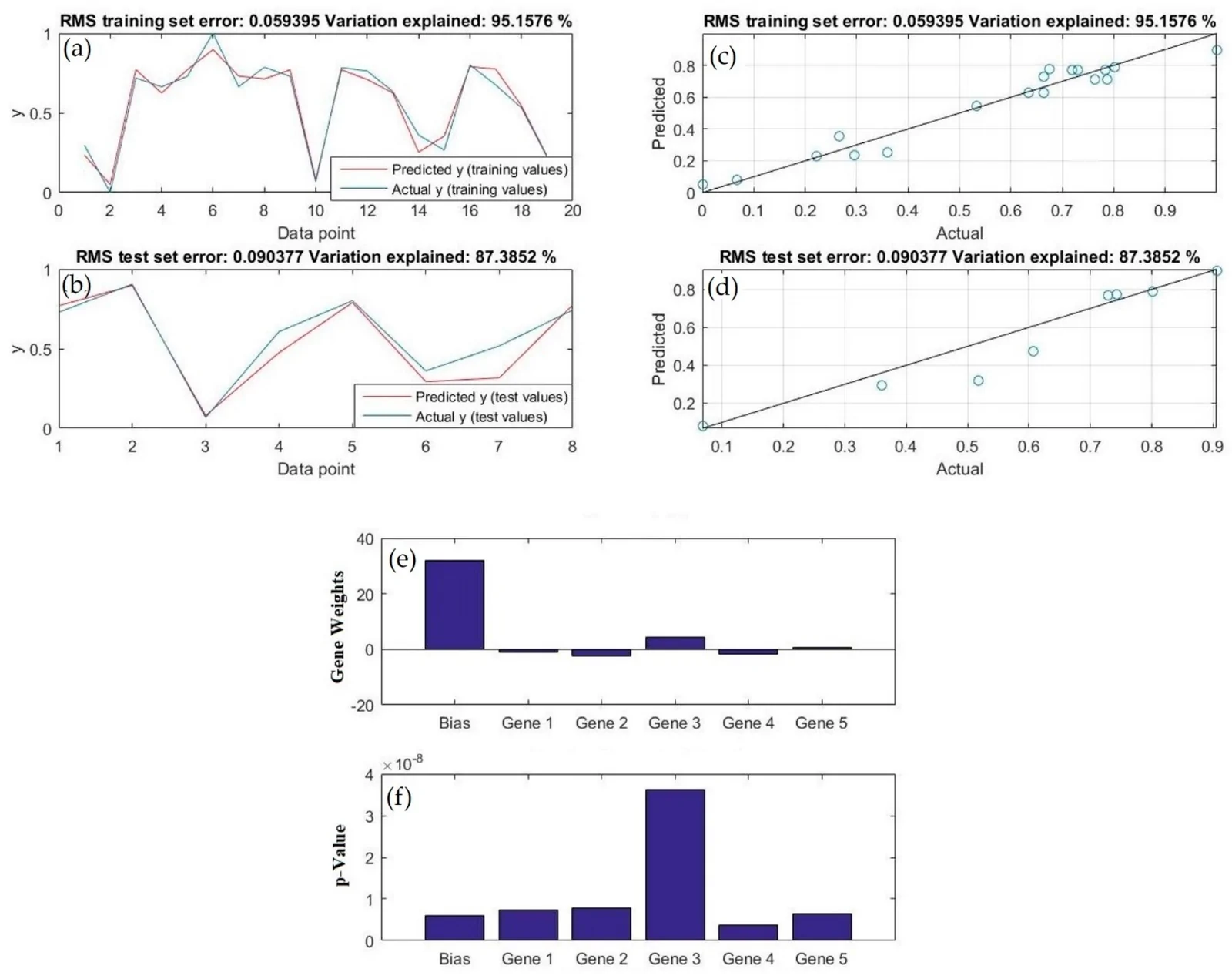
GPTIPS density outputs: response traces for (**a**) training and (**b**) evaluation observations; predicted-versus-observed values for (**c**) training and (**d**) evaluation observations; and (**e**) weighted gene contributions and (**f**) reported gene *p*-values. The plot labels “test” refer to the model-selection evaluation subset. Response coordinates are on the plotted transformed scale.

**Figure 8:**
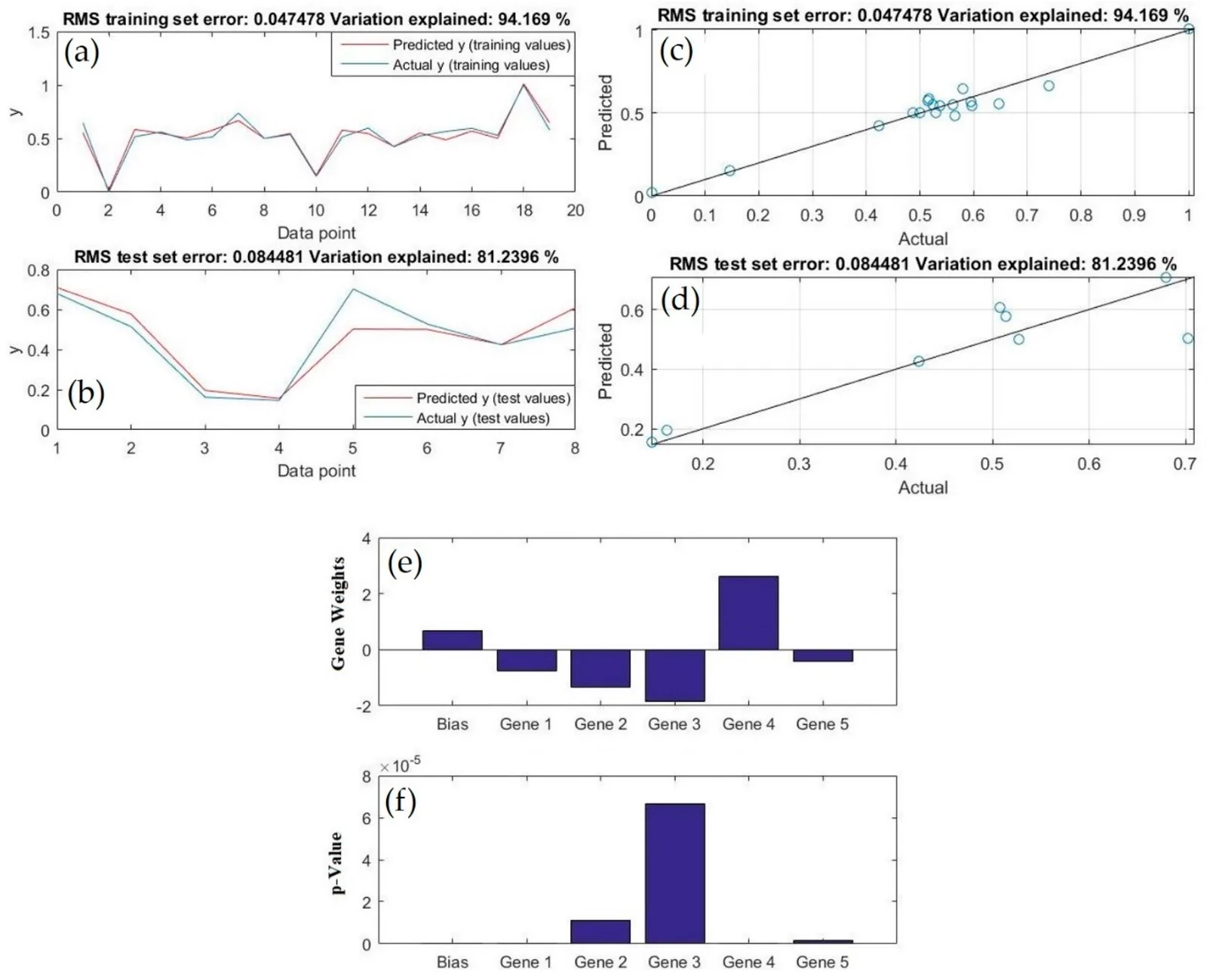
GPTIPS shrinkage outputs: response traces for (**a**) training and (**b**) evaluation observations; predicted-versus-observed values for (**c**) training and (**d**) evaluation observations; and (**e**) weighted gene contributions and (**f**) reported gene *p*-values. The plot labels “test” refer to the model-selection evaluation subset. Response coordinates are on the plotted transformed scale.

The symbolic-regression results are presented through the response-specific performance plots in Figure 6, Figure 7 and Figure 8. The plots use transformed response coordinates. Their numerical axes are therefore not interpreted directly as MPa, g/cm^3^ or percentage shrinkage, and no physical-unit RMSE is inferred from them.

Figure 6, Figure 7 and Figure 8 report training/evaluation variation explained of 97.697/89.5498% for strength, 95.1576/87.3852% for density and 94.169/81.2396% for shrinkage. The evaluation measure is highest for strength and lowest for shrinkage. All three evaluation values are lower than their training values, consistent with reduced fitted agreement outside the fitting subset. These percentages describe the displayed GPTIPS outputs; they are distinct from the RSM goodness-of-fit statistics in Appendix A and Appendix B.

Shrinkage is treated as a process response related to dimensional change. Greater shrinkage is not necessarily desirable because excessive or nonuniform contraction may impair dimensional tolerance and shape retention. Future multi-response optimisation should constrain shrinkage using a specified dimensional requirement while targeting adequate strength and density.

Symbolic regression provides an empirical description of the observed response patterns. Model fit alone does not identify the physical mechanism controlling densification or fracture.

The RSM statistics describe fits to the complete tabulated dataset, whereas the GPTIPS percentages describe training and model-selection subsets. These evaluation settings differ, so the numerical values do not establish superiority of one modelling method over the other.

### 3.5. Microstructural Characterisation

#### 3.5.1. EDX Elemental Distribution

**Figure 9:**
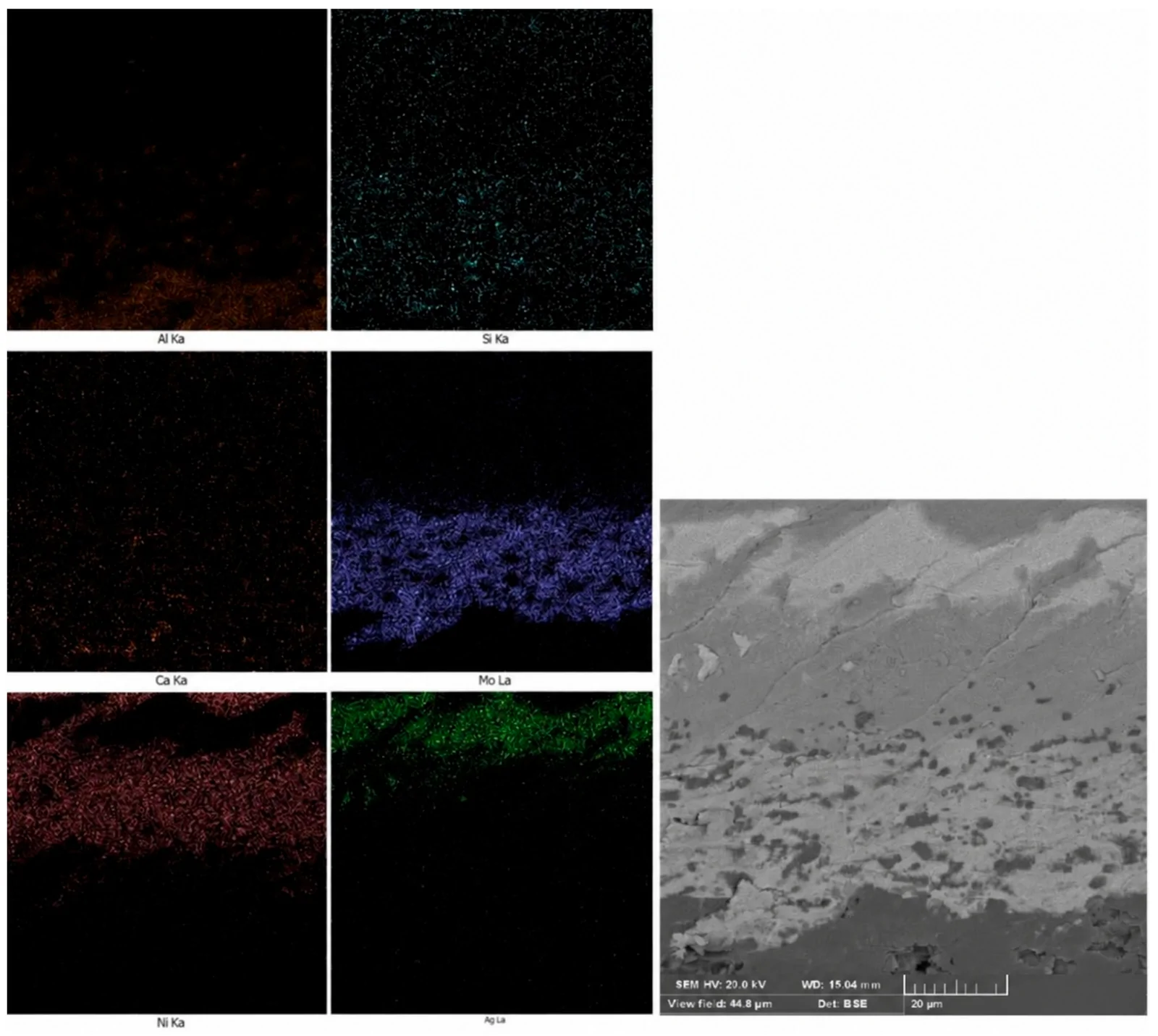
Original EDX elemental maps and associated BSE image of the cross-sectional region of specimen S2. The displayed channels are Al Kα, Si Kα, Ca Kα, Mo Lα, Ni Kα and Ag Lα. Channel labels are retained as recorded; the maps are qualitative and are not quantitative concentration profiles. The BSE image includes a 20 μm scale bar.

Figure 9 presents the original EDX maps and associated BSE image for specimen S2. The Al, Si and Ca channels show spatially heterogeneous signals across the imaged region. The Al signal is concentrated mainly towards the lower part of the field, whereas Si and Ca signals extend across a broader area. These maps provide qualitative information about spatial distribution; they do not establish a calibrated concentration gradient, diffusion distance or reaction-layer composition.

The EDX maps also contain signals labelled Mo, Ni and Ag, which are not constituents of the nominal CaO–SiO_2_–Al_2_O_3_ ceramic system. The Ag signal may be associated with conductive coating or silver-based mounting materials used during specimen preparation. Furthermore, spectral interference, background contributions or EDX peak misidentification may contribute to the additional elemental signals. As their precise origin cannot be confirmed from the available mapping results, these signals are not attributed to the ceramic composition or interfacial reaction products. The maps are therefore interpreted qualitatively and are not considered conclusive evidence of elemental interdiffusion or reaction-layer formation.

#### 3.5.2. BSE Microstructural Observations

**Figure 10:**
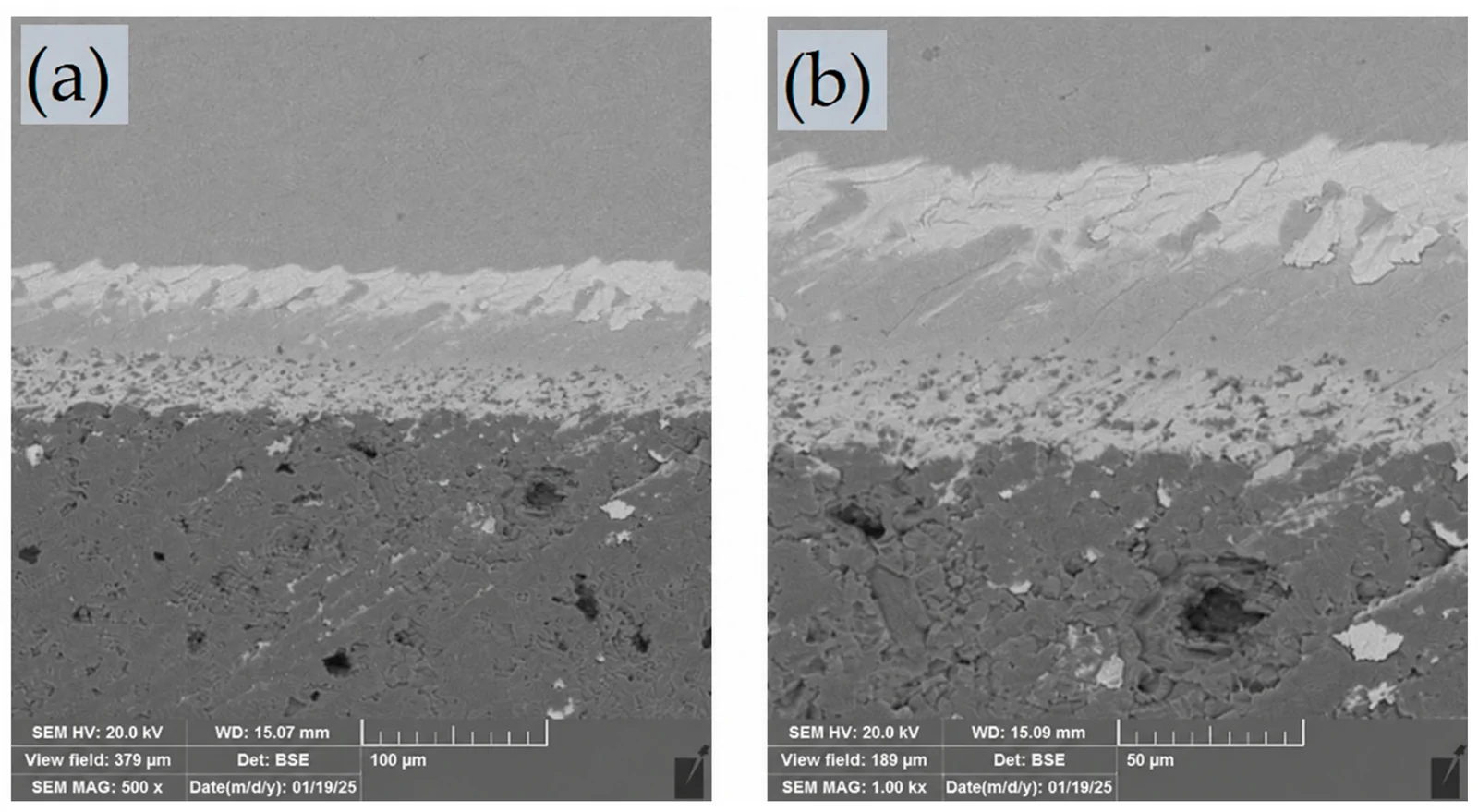
Original BSE cross-sectional images of specimen S2 at nominal magnifications of (**a**) 500× and (**b**) 1000×. Scale bars are (**a**) 100 μm and (**b**) 50 μm. Regions of differing contrast are described morphologically without assigning phase identity from brightness alone.

Figure 10 shows BSE cross-sections of specimen S2 at nominal magnifications of 500× and 1000×. Both images contain regions of contrasting grey level, including an irregular bright band and a heterogeneous lower region with dark features. The higher-magnification view resolves additional local variations in the band and surrounding material. The morphology is spatially nonuniform, so the images are not described as demonstrating a uniformly dense or defect-free interface.

The observed contrast is reported descriptively. Brightness alone is insufficient to identify alumina, a glassy phase or a reaction product, and the dark features are not assigned exclusively to porosity. The images therefore complement the specimen-level flexural results as qualitative structural observations; they do not measure interfacial bond strength or establish a diffusion-controlled consolidation mechanism.

## 4. Conclusions

This study applies symbolic regression and comparative response surface modelling to a 27-condition dataset for eutectic-assisted alumina ceramics, examining the relationships between processing parameters, flexural strength, density and sintering shrinkage.

The 27-condition analysis relates temperature, coarse-alumina fraction, lubricant content and additive content to strength, density and shrinkage. The highest tabulated strength is 173.52 MPa at 1550 °C, 25% coarse alumina, 1.5 wt% lubricant and 7.5 wt% additive. Shrinkage is treated as a dimensional response rather than a quantity to maximise.

Model specification strongly affects fitted agreement. Reconstruction of the published reduced-cubic strength and density models yields R^2^ values of 0.9825 and 0.9934, whereas the full-quadratic models give substantially lower adjusted R^2^. The 1533 °C processing condition is a constrained literature optimisation benchmark, distinct from the temperature of the maximum tabulated strength.

GPTIPS evaluation-subset variation explained is 89.55% for strength, 87.39% for density and 81.24% for shrinkage. These internal model-selection statistics support an exploratory description of the response patterns. They do not establish independent predictive validation or a causal microstructural mechanism.

Liquid-phase-assisted processing involves trade-offs between densification and the behaviour of residual grain-boundary phases. High-temperature creep, dielectric response and thermal-shock behaviour are relevant directions for further study, together with controlled post-sintering crystallisation. The present analysis does not assign a numerical service-temperature limit.

## Figures and Tables

**Table 1:** Nominal formulation of the ternary sintering additive.

Additive System	CaO (wt%)	Al_2_O_3_ (wt%)	SiO_2_ (wt%)	Basis for Composition Selection
CaO–Al_2_O_3_–SiO_2_	38	20	42	Composition investigated by Keramat and Hashemi [15]

**Table 2:** Sample designations and heat-treatment conditions investigated during preparation of the CaO–SiO_2_–Al_2_O_3_ additive.

Treatment Temperature (°C)	Sample Designation: 4 h Holding Time	Sample Designation: 8 h Holding Time
1200	G1	G2
1250	G3	G4

**Table 4:** Contextual benchmarks. Values are reported for different systems and test conditions and are not directly interchangeable.

System/Source	Processing	Reported Outcome	Comparison Limit
This Table 3, run 7	1550 °C; 7.5 wt% additive	173.52 MPa; 3.610 g/cm^3^	Tabulated maximum; same strength/density values as [15]; ROR specimen response
CuO–TiO_2_–Bi_2_O_3_/[22]	1200 °C; 90 min	97.63% relative density	Strength not stated in the consulted abstract; different chemistry
ZTA + CTN/[17]	1050 °C; intensive milling	235 MPa biaxial strength; 5.54% porosity	ZrO_2_ reinforcement and finer powders
Alumina + DAS infiltration/[18]	Glass distributed at boundaries at 1550 °C	525 ± 25 MPa flexural strength	Infiltration route; different glass and test conditions
Alumina + CTN/[19]	SPS: 750 °C, 60 MPa; pressureless: 1050 °C	Approximately 100% and 97% relative density, respectively	Pressure-assisted route is not a like-for-like furnace comparison

## Data Availability

Table 3 provides all 27 records used in the numerical analyses, with coded processing inputs and responses in physical units. Section 2.3 defines the conversions between coded and physical input values. The processing inputs, strength and density data originate from Reference [15], and source run identifiers are retained for traceability. Quadratic coefficients and ANOVA are provided in Appendix A. The extracted literature benchmark values and reconstructed reduced-cubic coefficients are identified in Appendix B.

## Appendix A. Quadratic Model Coefficients and ANOVA

The calculations use the 27 input–response records in Table 3 as unweighted observations without response transformation. The coded variables z1–z4 are defined in Section 2.3. The fitted form is y = β0 + Σβi zi + Σβii zi2 + Σ(i<j)βij zi zj. Each model has 15 coefficients and 12 residual degrees of freedom. Term rows report conditional drop-one-term sums of squares; these rows are not additive. Model SS equals corrected total SS minus residual SS. F-tests use the full-model residual mean square.

Pure error is estimated from the three centre-point runs (source run identifiers 6, 10 and 11 in Table 3), leaving two pure-error and 10 lack-of-fit degrees of freedom. This calculation assumes genuinely independent centre runs; if they are repeat measurements or derived values, the interpretation changes. Lack-of-fit tests with only two pure-error degrees of freedom have limited power; a nonsignificant test does not establish predictive adequacy. All values below are derived from rounded tabulated data. Numerical values are rounded for presentation only; the underlying calculations are unchanged.

**Table A1:** Flexural strength: conditional ANOVA. R^2^ = 0.612; adjusted R^2^ = 0.160; residual standard error = 18.5.

Source	df	SS	MS	F	*p*
Model	14	6490	463	1.35	0.302
z1	1	68.4	68.4	0.200	0.663
z2	1	188	188	0.550	0.473
z3	1	109	109	0.319	0.583
z4	1	9.34	9.34	0.0273	0.872
z1^2^	1	311	311	0.908	0.359
z2^2^	1	1640	1640	4.78	0.049
z3^2^	1	245	245	0.715	0.414
z4^2^	1	16.0	16.0	0.0466	0.833
z1z2	1	394	394	1.15	0.304
z1z3	1	3130	3130	9.16	0.011
z1z4	1	228	228	0.667	0.430
z2z3	1	362	362	1.06	0.324
z2z4	1	192	192	0.562	0.468
z3z4	1	0.0564	0.0564	1.65 × 10^−4^	0.990
Residual	12	4100	342	—	—
Lack of fit	10	3940	394	4.74	0.187
Pure error	2	166	83.1	—	—
Total	26	1.06 × 10^4^	—	—	—

**Table A2:** Density: conditional ANOVA. R^2^ = 0.594; adjusted R^2^ = 0.121; residual standard error = 0.0969.

Source	df	SS	MS	F	*p*
Model	14	0.165	0.0118	1.26	0.350
z1	1	4.42 × 10^−4^	4.42 × 10^−4^	0.0471	0.832
z2	1	0.00519	0.00519	0.553	0.472
z3	1	0.00263	0.00263	0.280	0.607
z4	1	0.00531	0.00531	0.566	0.467
z1^2^	1	0.00925	0.00925	0.985	0.341
z2^2^	1	0.0387	0.0387	4.12	0.065
z3^2^	1	0.00317	0.00317	0.338	0.572
z4^2^	1	2.88 × 10^−4^	2.88 × 10^−4^	0.0307	0.864
z1z2	1	0.00357	0.00357	0.380	0.549
z1z3	1	0.0794	0.0794	8.45	0.013
z1z4	1	1.16 × 10^−4^	1.16 × 10^−4^	0.0123	0.914
z2z3	1	0.0106	0.0106	1.12	0.310
z2z4	1	0.00439	0.00439	0.467	0.507
z3z4	1	0.00406	0.00406	0.433	0.523
Residual	12	0.113	0.00939	—	—
Lack of fit	10	0.111	0.0111	15.3	0.063
Pure error	2	0.00146	7.29 × 10^−4^	—	—
Total	26	0.278	—	—	—

**Table A3:** Shrinkage: conditional ANOVA. R^2^ = 0.408; adjusted R^2^ = −0.283; residual standard error = 3.49.

Source	df	SS	MS	F	*p*
Model	14	101	7.21	0.591	0.827
z1	1	36.6	36.6	3.00	0.109
z2	1	2.25	2.25	0.184	0.676
z3	1	4.08	4.08	0.335	0.574
z4	1	1.44	1.44	0.118	0.737
z1^2^	1	0.458	0.458	0.0375	0.850
z2^2^	1	8.88	8.88	0.728	0.410
z3^2^	1	4.06	4.06	0.332	0.575
z4^2^	1	6.84	6.84	0.560	0.469
z1z2	1	0.0306	0.0306	0.00251	0.961
z1z3	1	16.8	16.8	1.38	0.263
z1z4	1	1.68	1.68	0.137	0.717
z2z3	1	7.70	7.70	0.631	0.442
z2z4	1	0.185	0.185	0.0152	0.904
z3z4	1	0.126	0.126	0.0103	0.921
Residual	12	146	12.2	—	—
Lack of fit	10	143	14.3	8.50	0.110
Pure error	2	3.37	1.68	—	—
Total	26	247	—	—	—

**Table A4:** Quadratic coefficients. Strength is in MPa, density in g/cm^3^ and shrinkage in the reported percent units; coefficients use the coded inputs in Section 2.3.

Term	Flexural Strength	Density	Shrinkage
Intercept	151.0	3.498	28.88
z1	−1.688	−0.004292	−1.234
z2	−2.800	−0.01471	−0.3058
z3	2.132	0.01046	−0.4125
z4	0.6238	0.01488	−0.2450
z1^2^	−3.816	−0.02082	−0.1465
z2^2^	−8.753	−0.04257	−0.6452
z3^2^	−3.386	−0.01220	0.4360
z4^2^	−0.8647	0.003677	0.5660
z1z2	−4.962	−0.01494	0.04375
z1z3	14.00	0.07044	1.025
z1z4	3.774	0.002688	−0.3238
z2z3	−4.754	−0.02569	−0.6938
z2z4	3.467	0.01656	−0.1075
z3z4	0.05938	0.01594	−0.08875

## Appendix B. Extraction and Comparison of the Published RSM Results

The literature comparison uses Table 5 (p. 3164), Tables 6 and 7, the reduced-cubic expressions and Tables 8–10 of Keramat and Hashemi [15]. Strength, density and their four associated inputs were extracted for model reconstruction. The source optimisation predictions and confirmation measurements are reported separately in Table A7.

With the factor coding in Section 2.3, the retained basis is 1, z1, z2, z3, z4, z1z2, z1z3, z1z4, z2z3, z2z4, z3z4, z1^2^, z2^2^, z3^2^, z4^2^, z1z2z3, z1z2z4, z1z3z4, z2z3z4, z1^2^z2, z1^2^z3, z1^2^z4 and z1z2^2^. The physical response is the sum of each basis term multiplied by its coefficient in Table A5. This basis is taken from the published model specification, not selected anew by searching this dataset. Calculations use unweighted ordinary least squares on rounded published values. In Table A5, A = z1, B = z2, C = z3 and D = z4; the letter C in that table is the coded lubricant factor, not the physical coarse-alumina percentage.

**Table A5:** Reconstructed literature-model coefficients. Strength is in MPa; density is in g/cm^3^.

Term	Strength	Density
Intercept	151.0	3.498
A	−11.93	−0.05125
B	−8.348	−0.06275
C	2.938	0.01350
D	1.805	0.006750
AB	−4.962	−0.01494
AC	14.00	0.07044
AD	3.774	0.002688
BC	−4.754	−0.02569
BD	3.467	0.01656
CD	0.05938	0.01594
A^2^	−3.816	−0.02082
B^2^	−8.753	−0.04257
C^2^	−3.386	−0.01220
D^2^	−0.8647	0.003677
ABC	−8.858	−0.03144
ABD	−0.1469	0.009563
ACD	−4.727	−0.03081
BCD	−6.384	−0.03819
A^2^B	8.322	0.07206
A^2^C	−1.208	−0.004563
A^2^D	−1.772	0.01219
AB^2^	15.37	0.07044

**Table A6:** Model-level ANOVA for the reconstructed literature reduced-cubic fits. Published centre-point runs with source identifiers 6, 10 and 11 supply the pure-error estimate.

Response/Source	df	SS	MS	F	*p*
strength/Model	22	1.04 × 10^4^	473	10.2	0.018
strength/Residual	4	185	46.3	—	—
strength/Lack of fit	2	18.9	9.46	0.114	0.898
strength/Pure error	2	166	83.1	—	—
strength/Corrected total	26	1.06 × 10^4^	—	—	—
density/Model	22	0.276	0.0125	27.4	0.003
density/Residual	4	0.00183	4.59 × 10^−4^	—	—
density/Lack of fit	2	3.76 × 10^−4^	1.88 × 10^−4^	0.258	0.795
density/Pure error	2	0.00146	7.29 × 10^−4^	—	—
density/Corrected total	26	0.278	—	—	—

The strength fit has R^2^ = 0.983 and adjusted R^2^ = 0.886; density has R^2^ = 0.993 and adjusted R^2^ = 0.957. The models have four residual and two pure-error degrees of freedom. Their nonsignificant lack-of-fit statistics and high fitted agreement are interpreted within this limited design rather than as evidence of independent predictive accuracy.

For the source optimisation, Table 8 of [15] minimises temperature within 1450–1550 °C, holds coarse fraction within 25–75%, lubricant within 0.5–1.5 wt% and additive within 5.5–7.5 wt%, and maximises strength and density over the reported response bounds of 98.74–173.52 MPa and 3.197–3.610 g/cm^3^. Lower and upper weights are 1; importance is 3 except for additive content and density, for which it is 2. These are the source’s desirability settings, not newly selected objectives for the present experiments.

**Table A7:** Published optimum and confirmation comparison extracted from Tables 9 and 10 of [15].

Quantity	Published Prediction	Published Confirmation
Temperature (°C)	1533	1533
Coarse alumina (mass%)	25	25
Lubricant (wt%)	1.5	1.5
Additive (wt%)	7.5	7.5
Strength (MPa)	172.49	163.15
Density (g/cm^3^)	3.566	3.767

Confirmation minus prediction is −9.34 MPa for strength and +0.201 g/cm^3^ for density, calculated from the printed source values. These deviations quantify the published benchmark comparison and are not error estimates for GPTIPS.

## References

[1] Auerkari P. (1996). Mechanical and Physical Properties of Engineering Alumina Ceramics.

[2] Edmund J.M., Andersen C.E., Marckmann C.J., Aznar M.C., Akselrod M.S., Bøtter-Jensen L. (2006). CW-OSL measurement protocols using optical fibre Al_2_O_3_:Cdosemeters. Radiat. Prot. Dosim..

[3] Wu Y., Zhang Y., Choy K.L., Guo J. (2003). Liquid-phase sintering of alumina with YSiAlON oxynitride glass. Mater. Lett..

[4] Goswami A.P., Roy S., Das G.C. (2002). Effect of powder, chemistry and morphology on the dielectric properties of liquid-phase-sintered alumina. Ceram. Int..

[5] Svancarek P., Galusek D., Calvert C., Loughran F., Brown A., Brydson R., Riley F. (2004). A comparison of the microstructure and mechanical properties of two liquid phase sintered aluminas containing different molar ratios of calcia-silica sintering additives. J. Eur. Ceram. Soc..

[6] Thümmler F., Oberacker R. (2024). An Introduction to Powder Metallurgy.

[7] Mao H., Hillert M., Selleby M., Sundman B. (2006). Thermodynamic Assessment of the CaO–Al_2_O_3_–SiO_2_ System. J. Am. Ceram. Soc..

[8] Li J., Peng J., Guo S., Zhang L. (2013). Application of response surface methodology (RSM) for optimization of the sintering process of preparation calcia partially stabilized zirconia (CaO-PSZ) using natural baddeleyite. J. Alloys Compd..

[9] Sktani Z.D.I., Rejab N.A., Ratnam M.M., Ahmad Z.A. (2018). Fabrication of tougher ZTA ceramics with sustainable high hardness through (RSM) optimisation. Int. J. Refract. Met. Hard Mater..

[10] Das A., Sarkar S., Karanjai M., Sutradhar G. (2018). RSM Based Study on the Influence of Sintering Temperature on MRR for Titanium Powder Metallurgy Products using Box-Behnken Design. Mater. Today Proc..

[11] Rahaman M.N. (2017). Ceramic Processing and Sintering.

[12] Khajelakzay M., Bakhshi S.R. (2017). Optimization of spark plasma sintering parameters of Si3N4-SiC composite using response surface methodology (RSM). Ceram. Int..

[13] Chatterjee D., Oraon B., Sutradhar G., Bose P.K. (2007). Prediction of hardness for sintered HSS components using response surface method. J. Mater. Process. Technol..

[14] Elsen S.R., Ramesh T. (2016). Analysis and optimization of dry sliding wear characteristics of zirconia reinforced alumina composites formed by conventional sintering using response surface method. Int. J. Refract. Met. Hard Mater..

[15] Keramat E., Hashemi B. (2021). Modelling and optimizing the liquid phase sintering of alumina/CaO–SiO_2_–Al_2_O_3_ ceramics using response surface methodology. Ceram. Int..

[16] Searson D.P., Gandomi A.H., Alavi A.H., Ryan C. (2015). GPTIPS 2: An Open-Source Software Platform for Symbolic Data Mining. Handbook of Genetic Programming Applications.

[17] Garanin Y.A., Shakirzyanov R.I., Kaliyekperov M.E. (2025). Sintering of Alumina-Reinforced Ceramics Using Low-Temperature Sintering Additive. Crystals.

[18] Tang M., Zhu W., Zou H., Zu G., Han Y., Ran X. (2025). Fabrication of self-healing and high strength alumina/glass composite by infiltration route and its application for repairing cracks in dense ceramic. Ceram. Int..

[19] Stosz M., Kuglarz K., Longo F., Borgschulte A., Eriksson M., Johnsson M., Blugan G. (2026). Dense alumina by combining ternary sintering additives with spark plasma sintering at 750 °C. Ceram. Int..

[20] Fessler H., Fricker D.C. (1984). A Theoretical Analysis of the Ring-On-Ring Loading Disk Test. J. Am. Ceram. Soc..

[21] (2024). Standard Test Method for Monotonic Equibiaxial Flexural Strength of Advanced Ceramics at Ambient Temperature.

[22] Jiao R., Rong S., Wang D. (2022). Effect of dual liquid phase sintering aids on the densification and microstructure of low temperature sintered alumina ceramics. Ceram. Int..

